# Management of COVID-19 Pandemic Data in India: Challenges Faced and Lessons Learnt

**DOI:** 10.3389/fdata.2021.790158

**Published:** 2021-11-30

**Authors:** Jasmine Kaur, Jasleen Kaur, Ajay Singh Dhama, Vinit Kumar, Harpreet Singh

**Affiliations:** Division of Biomedical Informatics, Indian Council of Medical Research, New Delhi, India

**Keywords:** COVID-19, pandemic data, management of COVID-19 data, data management tool for COVID-19, webapi for COVID-19

## Abstract

COVID-19 is an ongoing pandemic, which has already claimed millions of lives worldwide. In the absence of prior information on the pandemic, the governments can use generated testing data to drive policy decisions. Thus, a one-stop repository is essential to ensure sharing of clean, de-duplicated, and updated records to all the stakeholders. In India, the government initiated the testing through a network of VRDLs headed by the Indian Council of Medical Research (ICMR). Initially, the generated data were captured and shared in Excel sheets. As the number of cases increased, there was a need for a data management system to ensure reliable and up-to-date data to drive policy decisions. Thus, the data management team at ICMR initiated the development of a national COVID-19 testing data management tool that is currently maintaining all the data in a central hub. The first version of the tool was released in March 2020 and was subsequently modified with the changing testing guidelines and strategies. On completing one and a half years of managing the data and collecting approximately 550 million records, the team analyzed the challenges faced and the strategies used to ensure a seamless flow of data to the system and its real-time analysis. In this study, the entire duration of the pandemic has been divided into four different phases based on the resourcefulness of the country. Since the pandemic is currently ongoing, this study can be useful for countries in a different phase of pandemic facing similar challenges.

## Introduction

The novel coronavirus disease COVID-19 is a global pandemic (Bedford et al., 2020). Since its inception in China around December 2019, it rapidly spread to more than 200 nations in less than 5 months (WHO, 2021). India reported its first case on January 30, 2020 (Andrews et al., 2020). Subsequently, the surveillance for COVID-19 cases was initiated by the government, along with various other agencies. Initially, COVID-19 was tested through only RT-PCR method and the generated testing data were collected and shared in Excel sheets. As the number of cases increased, there was a need for a data management tool to ensure reliable and up-to-date data are used to drive policy decisions. Thus, the ICMR data management team initiated the development of a national COVID-19 testing data collection and analysis tool. The tool has collected more than 550 million records and ensures sharing of clean, de-duplicated, and updated records with various stakeholders.

**FIGURE 1:**
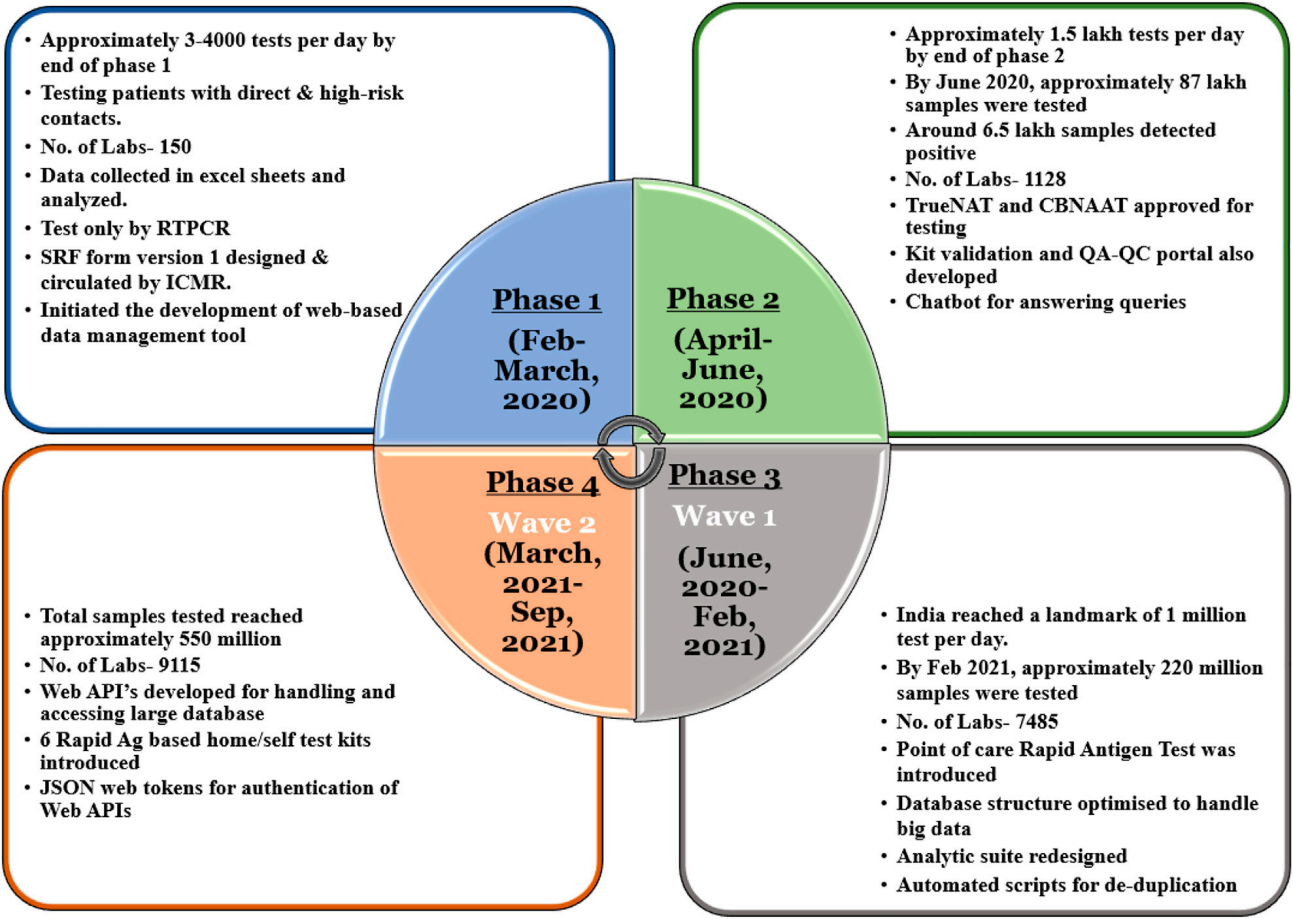
Phase-wise evolution of data management for COVID-19 in India.

**FIGURE 2:**
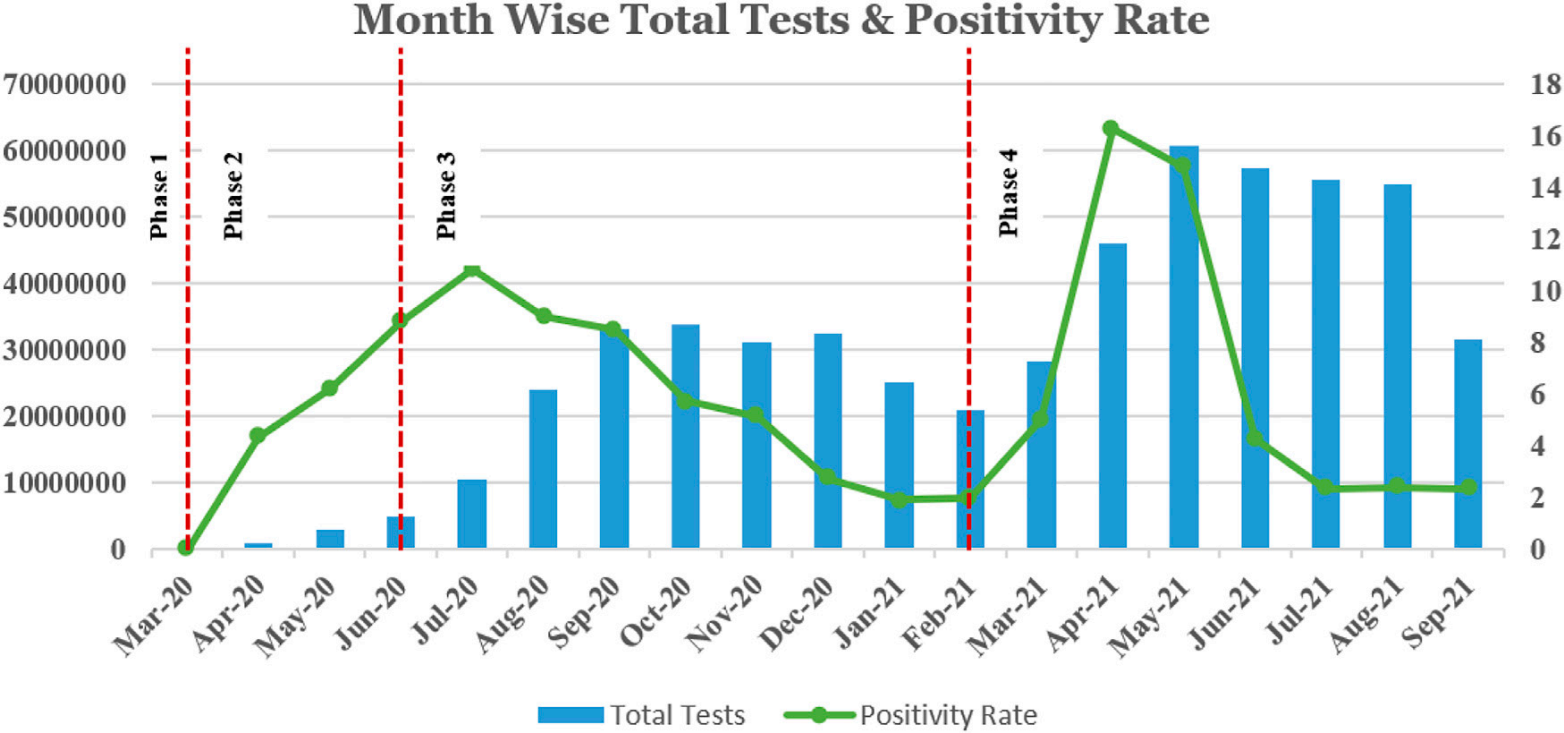
Month-wise total tests and positivity rate in India.

On completing one and a half years of managing the data and collecting approximately 550 million records, the team analyzed the challenges faced and the strategies used to ensure a seamless flow of data to the system and its real-time analysis. For this study, the entire duration of the pandemic has been divided into four different phases based on the resourcefulness of the country. For each phase, the paper discusses testing guidelines and strategies, data generated, challenges faced, and their implemented solutions (details in Figure 1 and Figure 2). Some of the challenges include transitioning from paper-based to online data collection tools, making adaptations based on the needs of various stakeholders/institutions, data reconciliation, and the ongoing challenge of big data management and analysis.

## Phases

### Phase 1 (Feb–March 2020)

*Scenario—*India’s laboratory surveillance for testing of COVID-19 is being led by the Indian Council of Medical Research (ICMR), which initiated RT-PCR testing in 78 selected national reference laboratories across the country (Abraham et al., 2020). In early 2020, there were limitations in the international supply of testing reagents, and guidelines were formulated by ICMR to ensure adequate testing of high-risk cases. On January 30, 2020, the first positive case was identified in Kerala, and more cases were subsequently reported in other parts of the country. On March 24, 2020, a nationwide lockdown was enacted to curb the spread of COVID (Balsari et al., 2020).

*Population tested and method used—*According to the ICMR’s initial guidelines, testing was recommended for symptomatic persons with a history of international travel or contact, as well as the healthcare workers treating patients with a severe acute respiratory infection. Furthermore, the strategy was revised to include patients admitted to the hospital with a severe acute respiratory illness. Among asymptomatic individuals, testing was recommended only for direct and high-risk contacts of a confirmed COVID-19 case (Rao et al., 2020). The initial rise in COVID-19-positive cases was majorly due to international travelers and their close contacts. The length of the infection was unknown. Thus, positive cases were quarantined and repeatedly tested every 24 h until two consecutive negative results were obtained.

*Data generated—*ICMR was constantly working towards augmenting the testing capacity, and by the end of March, approximately 150 labs were conducting 3–4,000 tests per day (ICMR,. 2021a) (ICMR,. 2021b).

*Challenges—*The initial data were collected and shared in Excel sheets. There was no standardized data collection format. Thus, a comprehensive analysis of these data was extremely difficult. ICMR designed and circulated a specimen referral form (SRF) to ensure uniformity in the information collected by all the laboratories (ICMR, 2021e). Also, with the increase in the number of laboratories and tests, managing Excel sheets became tedious and time-consuming. Hence, a web-based data management tool (based on SRF version 1) was designed and developed by the data management team at ICMR. The data entry form comprised of patient demographic details, sample details, and COVID test details along with the test results for other viruses. COVID-19 management involved the amalgamation of various agencies at national, state, and district levels. Moreover, the data circulating *via* calls, Excel sheets, etc. caused a huge discrepancy in the numbers reported at the national level. Thus, an entry into the online portal was strongly enforced. The Ministry of IT provided data entry operators at different sites, and the team provided training for online data entry at regular intervals through VCs. The real-time dashboards were designed and displayed to all the stakeholders.

### Phase 2 (April–June 2020)

*Scenario—*By this time, the coronavirus has spread rapidly across the country. Mortality increased from 100 confirmed COVID-19-associated deaths on April 5 to 1,000 by the end of the month (ICMR, 2021e). Many hotspots and containment zones were identified by the authorities. The testing methods such as TrueNAT and CBNAAT were approved to boost the testing capacity. Many companies started developing their own kits, which were then validated and approved by the ICMR. By June 2020, approximately 87 lakh samples were tested, and approximately 6.5 lakh samples tested positive (ICMR, 2021a) (ICMR, 2021b). The lockdown was implemented for the last time from May 30, 2020, and ease in restrictions was announced as a part of Unlock 1 from June 8, 2020 (Balsari et al., 2020).

The ICMR portal was being used to capture data across the country. However, some states began implementing their own data management applications and tools.

*Population tested—*This phase involved rigorously testing patients in hotspots and containment zones with influenza-like illnesses regardless of travel or contact history. Furthermore, the testing guidelines were added for asymptomatic pregnant women dwelling in cluster and confinement zones, major migrant gatherings, and evacuee centers, and presenting in delivery or likely to deliver within the next 5 days, although it was necessary to have a doctor’s prescription in order to be tested.

*Data generated—*By June 2020, the number of labs increased from 150 to 1,128, testing approximately 1.5 lakh samples per day.

*Challenges—*The SRF form underwent various revisions based on the testing guidelines, input from laboratories, and input from various stakeholders, which were then implemented in the online tool as well. The fields majorly captured involved patient demographic details, hospitalization details, sample details, patient category, symptoms and comorbidities, and testing details.

Additionally, an online tool was built where vendors can submit their kits in order to maintain consistency and transparency in the monitoring of kit validation. These kits were assigned to a validation center, where the vendor delivered the kits for validation and testing. The results were captured and displayed on the online portal (ICMR, 2021c). As more and more labs onboarded the ICMR list, quality assurance became a big concern. An online QA-QC portal (ICMR, 2021d) was developed to facilitate quarterly QC activities of the newly added labs.

The training and timely issue resolution became exceedingly challenging as the number of portals, laboratories, and connected human resources increased. Hence, the AI-assisted IBM-Watson chatbot was integrated into the portal (ICMR, 2020) (The Hindu, 2020). The chatbot was trained to answer basic data entry-related queries and to provide tutorials for all of the portal’s modules. All questions from various video conference calls were answered and fed into the bot on a regular basis.

During this period, another major challenge was transferring patients across the labs. As per the initial requirements, each lab had access to its own data. The expansion of temporary COVID hospitals and the establishment of small labs that could only do screening tests necessitated the transferring of patients between the sites. A global search option was enabled where labs could add follow-up information for patients initially tested in other centers. The automated reports and provision for labs to export their data in Excel were also enabled.

This movement created another major concern as the positive cases were entered by more than one lab and the patients would get tested outside their states as well. A data re-conciliation feature was enabled, allowing state and district officials access to a line list of cases in their area. The officials would constantly monitor the cases in their area and provide inputs for merging the records and modifications in the state or district of the patients.

Additionally, with the implementation of state portals, laboratories were overburdened with the data entry work. Subsequently, the number of errors and the backlog entries also increased. APIs were enabled for the transfer of data between ICMR and state applications. The API would be consumed by states to share the data directly in the ICMR portal. The comprehensive analysis was enabled for various stakeholders at the district, state, and national levels. This includes keeping track of total tests, positives, and positivity rates, as well as their testing capacity.

### Phase 3 (July 2020–Feb 2021)

*Scenario—*With the ease in restrictions by the government, people’s mobility expanded as did COVID’s. A point-of-care test, known as a rapid antigen test (RAT), was introduced to screen a larger group of population. With the introduction of the RAT, the testing increased enormously, and soon India reached a landmark of 1 million tests per day (Hindustan Times, 2020). Till February 2021, the total number of samples tested was 220 million. The number of laboratories increased to 7,485 including 3,500 logins provided to states for labs entering RAT data (ICMR,. 2021b) (ICMR,. 2021a).

*Population tested—*In this phase, testing on demand was incorporated, in contrast to earlier testing requirements for symptomatic persons with a doctor’s prescription. There was a mandatory requirement of testing for travel within and outside the country. As per the latest guidelines of ICMR, 17 categories for testing were devised.

*Data generated—*Presently, India is testing more than 1 million samples per day. This includes a screening of the general population along with testing in symptomatic, high-risk populations, and containment zones.

*Challenges—*With the onboarding of more labs and the introduction of the RAT, the size of the database increased enormously reducing the speed of data retrieval. The previous analysis became very slow. The concept of handling big data was used for database optimization. The analytic suite was completely redesigned and developed based on this concept. With an increase in workload on the same manpower, manual data reconciliation became extremely difficult. The automated scripts were implemented for duplicate identification and merging of records. This phase majorly involved transitioning from manual to automated systems for numerous tasks.

### Phase 4 (March 2021–September 2021)

*Scenario—*Till September 20, 2021, the number of samples tested has reached approximately 550 million. The number of laboratories increased from 7,485 to 9,115.

*Population tested—*There were not many changes in the testing guidelines. However, with the onset of the second wave of COVID-19 around April, many people were affected and, consequently, the number of samples tested increased drastically. This also initiated screening of the general population along with testing in symptomatic, high-risk populations and containment zones.

*Data generated—*The increase in cases in the country began in mid-March 2021 with the second wave and escalated in April 2021. COVID-19 cases surpassed 15.9 million as of April 23, 2021, with 185,000 mortality (Jain et al., 2021).

*Challenges—*In comparison to the first wave, the second wave moved at a breakneck speed. The ICMR’s COVID-19 testing database expanded significantly in size during the second wave, slowing down the fetching of state- and district-specific data. Therefore, during this period, the major challenge was ensuring smooth data retrieval for contact tracing and hotspot identification. To enable this, web-APIs were implemented. REST (REST, 2021) is a common approach for enabling the connectivity between a client and server using the HTTP protocol and a JSON exchange format (JSON, 2021a). The COVID data-API based on the REST approach was developed to download data by the states based on various filters. The data-API has been designed and developed by using standard HTTP response and human-readable URLs/endpoints. The downloaded data and input parameters are both in standard JSON format. The data-API can be consumed by other websites, as it has a cross-origin resource sharing option enabled. The data-API is authenticated *via* the JsonWebToken (JSON, 2021b) to authenticate a client. The web API server requested cvanalytics login credentials and used JsonWebToken authenticator to verify the credentials for an authorized user. The developed data-API works in a stateless mode, enabling each request to be executed independently from others without the requirement for the state to be retained on the server.

## Discussion, Conclusion, and Future Work

The course of the COVID-19 pandemic elucidated the vital role played by data in guiding effective decisions to save communities and economies worldwide. With no prior knowledge of the virus, the course of the disease, and its spread, data were the one reliable source used by countries to guide the decision-making process and verify the plan of action before it is committed. A one-stop data repository is required to ensure the sharing and analysis of clean and reliable data. The data repository also ensures the dissemination of correct information to the public. In India, the national database was developed and is being managed by the data management team at ICMR. This paper describes the challenges faced by the team and the strategies used to ensure a smooth flow of data to the system. The database also ensures the dissemination of correct information to the various stakeholders working on different aspects of the pandemic management.

Once we have a comprehensive data repository, various studies have shown the importance of the predictive analysis to forecast outbreaks (Iwendi et al., 2020) (Dhamodharavadhani and Chatterjee 2020) (Sujath and Chatterjee,. 2020) (Khan et al., 2021) (Khajanchi and Sarkar 2020). The epidemiological modeling techniques can be used for better understanding the virus patterns, developing the control methods for quickly growing infectious diseases, and reducing the basic reproduction number R_0_. The Statistical Neural Network models can also be applied to the comprehensive data collected in the ICMR central hub/one-stop repository to estimate the future COVID-19 death cases and reduce the mortality rate in India. Future work involves converting it to a standard-based customizable system that can be used for the management and analysis of any future epidemics.
